# Economics of the community mechanism

**DOI:** 10.1007/s42973-022-00113-2

**Published:** 2022-04-18

**Authors:** Masao Ogaki

**Affiliations:** Faculty of Economics, Keio University, 2-15-45 Mita, Minato-ku, Tokyo, 108-8345 Japan

**Keywords:** Community mechanism, Altruism, Reciprocity, Trust, Virtue, Eudaimonia, A10, D01, D04

## Abstract

This paper discusses the importance of the community mechanism that complements the market and power mechanisms in an economic system during an era of crisis, defined as a mechanism for resource allocation by which at least one person proposes voluntary cooperation, and the proposal is not rejected. While this community mechanism can function alongside *homo economicus* in win–win situations, it can be further activated with social preferences for altruism and reciprocity and with norms or worldviews that encourage cooperation. Other factors that relate to these include the character strengths that contribute to community and society known as virtues, with the concept of wellbeing related to virtues being known as eudaimonia. Some aspects of the acquisition of virtues can be viewed as changing preferences, and there is empirical evidence suggesting changes in trust relate to changes in preferences. Leadership is an example of the virtue of justice, and servant leadership seems important for the community mechanism, as does perspective taking. For evaluating policies, normative economics based only on consequentialism may not be sufficient, and virtue ethics seems essential when the community mechanism is important.

## Introduction

There are three major mechanisms at work in the economic system. These are the *market mechanism* (comprising the price mechanism and the competition mechanism), the *power mechanism* (a mechanism that can, for example, coerce people to pay taxes because of the power of the legal system), and the *community mechanism* (a mechanism in which at least one person proposes voluntary cooperation and is not rejected).

We discuss the definition of the community mechanism in detail in Sect. 3. A few key points to start. First, the community mechanism is intended to be distinct from the remaining two mechanisms, even though it can be combined with both or one of the two other mechanisms. Second, its main use is in what might be called the community sector of families and nonprofit organizations, but it is also used in the market sector of profit organizations, and the public sector of state and local public bodies.

As an example, consider the Japanese Economic Association (JEA). This paper is based on the presidential address given at the Fall Meeting of the JEA in 2021, which was the first hybrid conference of the JEA given the COVID-19 pandemic, consisting of both online and face-to-face meetings and building upon the JEA’s Spring Meeting in 2020, which was its first online conference. COVID-19 produced many challenges never experienced before by the JEA as an academic association, but the chairs of the steering and program committees and the directors in charge of the conference, and many other association members volunteered their time and efforts to address these and proceed with the conference. This shows the *community mechanism* at work.

In addition, without the administrative work of the JEA office workers who are not association members, activities such as the conference would not be possible. So, the *market mechanism* in the form of the labor market is also at work here. Lastly, in the poster session for the online conference, there is always the possibility that the presenter and the audience will be one-on-one, and it then becomes more important to prevent harassment in this situation. So, there are many activities relating to the law, such as the appointment of the first Anti-Harassment Committee at the 2020 Fall Conference, and the appointment of the new committee in charge of legal affairs in May 2021. This shows the *power mechanism* at work.

We typically consider that the three main sectors of the economy, being the market, public, and community sectors, correspond in turn to the market, power, and community mechanisms in the sense that it is in only one of each that one of the three mechanisms is at work. At first, this seems to hold for the JEA, which is neither a for-profit company in the market sector nor a government agency in the public sector but lies in the community sector and is based on the workings of the community mechanism by many volunteers. However, the association not only uses the community mechanism, but also mixes the market and power mechanisms with it to carry out projects for the purpose of the association. For-profit corporations in the market sector and national and local governments in the public sector also use a mixture of the three mechanisms, so it is necessary to distinguish between the three sectors and the three mechanisms. In this paper, I discuss how any group of two or more persons can use a mixture of the three mechanisms and how these mechanisms are and should be combined. The market mechanism, the power mechanism, and the community mechanism are all tools in their own right and can be used for good or evil.

The remainder of the paper is organized as follows. Section 2 discusses the importance of the community mechanism and its relationship with the stage of economic development. In low-income countries, the community mechanism plays an important role, but as economic development progresses, the community mechanism loses its importance. However, as economic development progresses further, periods of crisis arising from environmental problems and a declining birthrate and aging population will occur. Even in high-income countries, it will be difficult to respond to these challenges with the market and power mechanisms alone, and the community mechanism will once again become important.

Section 3 defines the community mechanism using the story of Robinson Crusoe and Friday. A definition of the community mechanism is proposed as a mechanism in which at least one person proposes voluntary cooperation that is not rejected. Sections 4 and 5 provide examples of how this community mechanism can be captured in traditional economics, which assumes selfish and rational economic agents (*homo economicus*), and in behavioral economics, which does not. The following sections consider the relationship between the community mechanism and various concepts: virtue and eudaimonia in Sect. 6, endogenous preferences and trust in Sect. 7, leadership in Sect. 8, and perspective taking and community mechanisms in Sect. 9.

Together, these sections provide the background for normative economics, which explores how scarce resources should be allocated, as opposed to positive economics, which explores how scarce resources are allocated. Section 10 then presents an analytical framework that introduces virtue ethics, an ethical approach that emphasizes virtue, which can play a major role in the development of the community mechanism into normative economics. It also presents an application of normative economics to the problem of childcare outsourcing. Finally, Sect. 11 presents the conclusion and future research direction.

## An era of crisis and the community mechanism

Sandel (2009) describes how a gas station in Orlando, Florida, sold $2 bags of ice for $10 after the devastation of Hurricane Charley in 2004, and how a 77-year-old woman who evacuated with her elderly husband and disabled daughter explained that she was charged $260 for a motel room that normally costs $40 a night. In these examples, there is an efficient market mechanism at work in the sense that there is no need to wait in line. However, the state of Florida has a law that prohibits price hikes, and one motel lost a lawsuit and needed to pay a total of $70,000 in fines and compensation (an example of the power mechanism).

By contrast, in the aftermath of 2011’s Great East Japan Earthquake, people lined up neatly even in front of half-empty stores, prompting the English-speaking Internet community to remark that the Japanese were calm and wonder if they would be able to do the same in a Western country following a similar disaster. Many stores did not use the market mechanism to raise prices but instead the community mechanism.

In the event of a catastrophe, it would be advisable to use all three mechanisms together, as each has their own advantages and disadvantages. With the power mechanism, taxes are used to distribute daily necessities free of charge, but this can lead to long queues. Alternatively, with the community mechanism stores will sell daily necessities without raising their prices, but the queues will be longer if they do not “raise prices on the fly” in response to the trust of neighboring communities. Lastly, the market mechanism raises the prices of daily necessities so that there is no queue. Accordingly, if a mother needing water for her baby immediately has the money and her baby’s life is at risk if she waits in line, she may still be willing to buy even if there is a store that is raising prices.

Currently, the world is experiencing large-scale disasters, pandemics of infectious diseases, and environmental problems. In addition, there is a heightened risk of crisis because of the declining birthrate and aging population in many countries around the world. For instance, there is a potential governmental financial crisis in Japan if real interest rates increase worldwide.

We cannot rely on the market mechanism alone to respond to crises. One important reason is that the increase in the number and population share of the elderly who cannot effectively use market mechanisms alone due to the natural decline of cognitive abilities and dementia caused by aging.^1^ Another reason is that the market mechanism may not be helpful in some urgent situations. For example, when there is an earthquake, imagine a situation in which only cash payments are possible, and you do not have cash. Likewise, relying solely on the power mechanism by the state to supplement the market mechanism will accelerate the government financial crisis caused by the declining birthrate and the aging population. There appears no other way but to use the community mechanism in conjunction with the other two mechanisms.

The community mechanism is important in low-income countries, while its importance declines as economic development begins. However, Ogaki and Ohtake (2019) predict that the importance of the community mechanism will again increase when the birthrate starts to decline and the population starts to age because it will be difficult to respond to these challenges with the market and power mechanisms alone.

Thus, the importance of the community mechanism is expected to rise again in the era of crisis, but the importance of understanding how the community mechanism works with the market and power mechanisms has been recognized for a long time. One reason is that continued increase in female labor participation means that the care work such as caring for children, the elderly, and the ill has shifted away from women in families to paid work (Folbre, 2001; Folbre & Nelson, 2000). Another reason is that the community mechanism in the gift exchange element is often important in worker–employer relationships. Cooper and Kagel (2016) survey the related and large experimental economics literature.

## Definition of the community mechanism

How should we define the community mechanism? Of course, how we define it will depend on the purpose of the research. In this section, we consider a definition for research purposes corresponding to times of crisis. Hayami (2009) introduced the concept of the community mechanism and defined it as a mechanism that guides community members to voluntary cooperation based on intensive social capital. He then divided the economic system into three main categories: the community mechanism, market mechanism, and state mechanism. While there have been other approaches that divided the economy into community, market, and state (e.g., Bowles & Gintis, 2002; Hayami, 1989), I would like to consider what, if any, significance there is in adding the word “mechanism.” Later, Ogaki and Ohtake (2019) defined the concept of community mechanism following Hayami (2009), but in their definition they introduced the role of human capital and spiritual capital as well as social capital. In addition, and unlike Hayami (2009), they defined the public mechanism used by the public sector, including local governments, as one of three mechanisms alongside the community mechanism and the market mechanism.

It is also possible to think of the economy in terms of sectors, such as the community sector, the market sector, and the state (or public) sector. Rajan (2019) identified the market, state, and community as the three pillars of the economy, with the third pillar being the local community. However, just as the JEA used a mixture of the three mechanisms in responding to COVID-19, there is the question of how a single individual, group, or sector can and should use a mixture of the three mechanisms. Thinking in terms of mechanisms rather than sectors allows us to better analyze this issue.

Because the three mechanisms are at least implicitly meant to be distinct from each other without overlap, the definition of the community mechanism is affected by how we define the other two mechanisms. When we define the state mechanism as in Hayami (2009) and the public mechanism as in Ogaki and Ohtake (2019), the definition of the community mechanism is affected by sectors. By defining the power mechanism without any reference to a sector, we can free ourselves from the sectors for a new definition of the community mechanism. For this reason, we reconsider the definition of the community mechanism by contemplating a basic model of two persons.

In macroeconomics, the most basic model is the so-called Robinson Crusoe economy of a representative individual, which assuming an aggregation theorem^2^ holds then extends to the economy level of many individuals. In Daniel Defoe’s novel Robinson Crusoe, Robinson, who has been living on his own for 25 years on an uninhabited island, rescues a prisoner of war (who he names Friday) about to be killed and eaten when the cannibalistic inhabitants of a neighboring island come ashore. To think about a basic model for the three mechanisms, we can refer to this story of Robinson and Friday, who are completely different in language and culture, but meet in a crisis where they live alone on an island where it is difficult for them to survive.

Robinson has guns, which Friday does not even know how to use. Robinson initially threatens Friday with a gun to get him to obey his orders. Robinson also uses his gun to force Friday to obey his social norm that cannibalism is a bad thing. This is a power mechanism. At first, Robinson is careful to devise a way to prevent Friday from attacking him while he was sleeping. However, Friday has a strong reciprocity nature and sees Robinson as a lifesaver. This can be thought of as the emergence of a unilateral sense of community, where Friday is aware that Robinson and he belong to one community. Friday then acts out of a desire to repay the favor. This is a unilateral and voluntary cooperative action based on Friday’s reciprocity nature, and because Robinson does not reject Friday’s offer of cooperative behavior for the common purpose of surviving on an isolated island, a two-way community mechanism begins to work.

Realizing this reciprocity, Robinson begins to trust Friday (the development of a community mechanism based on increasing trust with Robinson’s more accurate belief in Friday’s trustworthiness). Robinson also realizes that he needs to not only force Friday to stop cannibalism through social norms, but also teach him the taste of goat meat so that he changes his preferences (a change in preferences better suited for community cooperation through leader intervention). Robinson also teaches Friday English (a development of the community mechanism based on increased communication skills). Later still, Robinson introduces Friday to Christianity (God, who created the heavens and the earth, is in the heavens, not just living in a mountain on a nearby island and prophesying to those who come to the mountain), answers Friday’s questions (“If God is omniscient and omnipotent, why doesn’t he just kill the devil right away?” etc.), and begins to share his own values and ethics (a community mechanism based on a shared worldview).

Eventually, Friday expresses to Robinson his desire to return to his own island, and Robinson suspects that Friday originally intended to kill him. When Robinson is convinced that this suspicion was wrong, he thinks that he should have trusted Friday, who had come to share the same moral values. This can be thought of in political scientist Uslaner’s (2008) view of trust as a moral value (a trust that goes beyond the rational belief in trustworthiness from personal experience, etc., and accepts another person into a moral community).

Although the public sector does not appear in Robinson and Friday’s story, we can define the power mechanism as a mechanism for the allocation of scarce resources (goods, services, etc.) by power that can be coerced without the need for consent. By this definition, the coercion of tax payments by the police force is a power mechanism in which the public sector uses public power. Just as Robinson used the power of the gun to coerce, the coercion of behavior in accordance with social norms using bad language in the mass media, social networking sites, etc., can also be defined as a power mechanism. And as before, the market mechanism is a mechanism for allocating resources by voluntarily agreeing to terms of exchange such as price and quantity.

Based on the story of Robinson and Friday, we can define the community mechanism as a mechanism for resource allocation by which at least one person proposes voluntary cooperation and the proposal is not rejected that are not the market or the power mechanisms.^3^ If defined in this way, trust, reciprocity, altruism, or any social capital is not the minimum requirement for a community mechanism to work.^4^ Even if there is no trust or compassion, if a win–win cooperative relationship is proposed for substantial benefits and it is not rejected, then the community mechanism begins to work. Using this definition, we refer to the group of people in which the community mechanism is working as a *community*. In this sense, the term *community* includes commercial enterprises, where the market mechanism is primarily at work, and the state, where the power mechanism is primarily at work. To see how the community mechanism works, it is important to examine how it works for different groups. For this purpose, we consider the family, the nation, and the international community.

First, let us consider the family as a community and think about the functioning of the community mechanism in the family. When a newborn baby is born, there can be special situations and environments in which parents cannot feel sincere affection for their own baby. Even in this case, the community mechanism starts to work if the service of nurturing is provided to the newborn and the newborn does not refuse (for example, because of illness). The community mechanism may be deepened as the parents and the child develop trust and sympathy for each other in the process of parenting.

The first unified approach to the economics of the family was based on Becker’s (1973, 1974, 1981) unitary model, in which the family is a single, unified decision-making entity. The standard model that justifies the unitary model is Becker’s (1974) model in which the head of the household has altruism toward other family members. Subsequently, many approaches to nonunitary models have been applied without any altruism between family members, and cooperative and repeated games have been used in theoretical and empirical studies (for a description of these approaches, see, e.g., Browning et al., 2014).

Next, let us consider the functioning of the community mechanism in a nation, now considering the nation as a community. When you are born, you cannot choose which country you will be a citizen of. We cannot say that we have agreed to obey the laws of the country, so cooperative behavior to obey the laws is enforced by the power mechanism. However, if you are given the right to renounce your nationality once you become an adult, you agree to obey the laws of the country, and the community mechanism starts working alongside the power mechanism.

Finally, thinking of the international community, if the community does not use the power mechanism with armies, then it will use the market mechanism (international trade and financial markets) and/or the community mechanism (international help based on gratitude to the country that received aid during a major disaster, international cooperation based on trust, etc.).

## Selfish and rational economic man and the community mechanism

My impression of the term *community mechanism* used to be that trust, reciprocity, and altruism are necessary for it to work. With this impression, among the theories of traditional economics, which assume each agent is a selfish and rational economic man (*homo economicus*), only the theories of repeated games, economics of families, and theories of public and club goods, including an application of the club good theory to religious organizations such as by Iannaccone (1992), used to seem relevant for the community mechanism. If we define the community mechanism as a mechanism for allocating resources by which at least one person proposes voluntary cooperation, and the proposal is not rejected, however, then reciprocity and altruism are not necessary conditions for the community mechanism to work. One of the advantages of such a definition is that it allows us to directly apply a wide range of traditional economic theories and findings on cooperation, norms, contracts, etc., from empirical studies based on these theories for the purpose of research into the community mechanism. In this section, I briefly consider such applications.

First, let us take the ultimatum game, which has been the subject of much theoretical and experimental research, from the perspective of a game that can be interpreted as being about the minimum necessary condition for the community mechanism to propose and accept voluntary cooperation. There are many variations of this game, but typically the proposer among two randomly matched anonymous individuals receives an initial amount of money (e.g., 1,000 cents). The proposer offers (1000−*x*), where *x* is the amount to be allocated to the responder (in increments of one cent, such that 0, 1, 2, …, 1000 can be offered). The responder decides whether to accept or reject the offer. If the offer is accepted, the proposer retains 1000−*x* cents, and the responder is paid *x* cents. If the offer is rejected, both parties receive zero cents.

If the responder accepts the offer, no matter how much the offer is, a cooperative relationship is established between the two parties, which means that the community mechanism is at work, and the allocation of resources is Pareto efficient. If we assume that both players are selfish and rational economic men, and consider only pure strategies, the subgame perfect equilibrium will be reached only in the two cases of an offer of zero cents and acceptance and an offer of one cent and acceptance.

In contrast to this theoretical prediction, in actual experiments, as shown by the meta-analysis by Oosterbeek et al. (2004), responders on average reject about 16% of offers, and proposers offer on average about 40% of their initial holdings. Subgame perfect equilibria do not explain these experimental results. However, Gale et al. (1995) build a model that introduces noise and learning and explain their experimental results by showing that the learning process leads to a Nash equilibrium that is not a subgame perfect equilibrium.

Although it is standard for sequential games to be analyzed in subgame perfect equilibrium, it is possible to explain the experimental results of ultimatum games in this way with a model that assumes selfish and rational economic agents. To simultaneously explain the experimental results of other games, such as dictator games, however, it seems to us that a behavioral economics model without this assumption, as presented in the next section, would be more natural, although it is not easy to reject this assumption when we consider various factors. This is just one example.

Next, let us consider the case where the efficiency principle holds for a group of two or more people in which the community mechanism may operate. Milgrom and Roberts (1992, p. 24) explain this principle as follows: “If people are able to bargain together effectively and can effectively implement and enforce their decisions, then the outcomes of economic activity will tend to be efficient (at least for the parties to the bargain).”

For example, in the setting of a nonrepeating prisoner’s dilemma game, if the two prisoners are free to negotiate through secret means of communication, and if there is an ironclad rule that if they break their agreement, they will be killed as soon as they leave the prison, they will choose cooperation. So even if the prisoners do not have altruism and do not trust each other, if they can negotiate freely and the ironclad rule provides security, they will choose to cooperate in such a way that satisfies the efficiency principle. For this reason, the findings from cooperative game theory may be useful for the purpose of studying the community mechanism.

From this point of view, solutions to various cooperative games, such as von Neuman and Morgenstern’s (1944) stable set and Nash’s (1950) bargaining solution, can be regarded as theoretical predictions for the case where the community mechanism works. The Nash program, in which the solution of a cooperative game becomes the solution of an appropriate noncooperative game (Nash, 1953), also bridges cooperative game theory and noncooperative games for the analysis of the community mechanism. Similarly, Brandenburger and Nalebuff’s (1996) argument that for-profit companies can maximize profits by not only competition (the market mechanism) but also cooperation (the community mechanism) using cooperative game theory can be seen as a case of combining the market and community mechanisms.

Next, even in a noncooperative game, cooperation becomes easier if there is a long-term relationship in a repeated game. For example, if the choice not to cooperate in an infinitely repeated prisoner’s dilemma game is followed by temporary or permanent exclusion from the cooperative relationship, then each subject has an incentive to cooperate. If we consider a repeated game in which two people are randomly matched within a community in each period, there is a difference between personal enforcement, in which the betrayed person does not cooperate with the betrayer in the future, and community enforcement, in which members other than the betrayed person do not cooperate. In one of Kandori’s (1992) settings, the norm of contagious strategy emerges in which trust is not placed in the individual but in the community, and once one person is betrayed, that person will not cooperate with anyone thereafter. In Kandori’s (1992) alternative setting, cooperation is maintained by labeling the betrayer. This label is interpreted as equivalent to a reputation, membership, or license in a real community. Dal Bó and Fréchette (2018) provide a survey of the experimental economics results of repeated games including community enforcement.

Many corporations and nonprofit organizations publish their purpose on the Internet and in their articles of incorporation. The objectives of the members of a community may often be different from the objectives of the community, but why do many communities publish their *community objectives* even when it is not required by law? In game theory, focal points play an important role when there are multiple equilibria (Schelling, 1960), so one explanation is that the community’s purpose provides a focal point. In addition, when commitment is important, the announcement of the community’s purpose may play a role in helping commitment.

## Community mechanisms in behavioral economics

Although various definitions are possible for behavioral economics, we adopt Ogaki and Tanaka’s (2017) definition as “the study of economics that does not rely on the assumptions of selfish and rational economic man.” The theory of incomplete contracts, which assumes bounded rationality, is explained in the textbook on the economics of organizations by Milgrom and Roberts (1992).

“Selfishness” in this definition means that one is only interested in one’s own payoffs, consumption, and leisure, and when one chooses to follow a norm, one does not derive utility from it per se, but only from what will happen to one’s consumption, leisure, and gain in the future if one does not follow the norm. First, let us consider what types of economic models with economic agents who are not selfish are candidates for explaining experimental results of different variations of the dictator game.

A dictator game is a modification of an ultimatum game in which the responders are not given a veto. The dictator unilaterally divides the initial holdings. A selfish and rational economic man would monopolize the initial holdings. In actual experiments, the dictator typically gives about 30% of the initial holdings (Engel, 2011). As candidate models to explain this result, we consider a model with social preferences (or other-regarding preferences), a model of norms, and a model of worldviews.

To explain the results of the dictator game and the ultimatum game, it is natural to assume that the preferences reflect some consideration of fairness, but the results of the market experiments with the double auction can be explained by a supply and demand model with selfish and rational economic men. In the late 1990s, the inequality aversion models of Fehr and Schmidt (1999) and Bolton and Ockenfels (2000) were able to explain the results of many of these experiments in a unified way. In these models, utility declines when there is self-centered inequality, that is, inequality with others in comparison to oneself, and rises when one’s own gain increases when inequality is constant. These are models of outcome-based social preferences that only depend on outcomes. Alternatively, there is an intention-based social preference model, such as Rabin’s (1993) model, in which one’s utility rises as others’ gains increase if one believes that they have kind intentions toward one, and one’s utility declines if one believes that they have hostile intentions toward one. Conversely, if one holds the belief that others have hostile intentions, one’s utility will fall as their gains rise.^5^

Several related economic experimental studies since the mid-1990s are reviewed by Cooper and Kagel (2016). In List (2007) and Bardsley (2008), the option to take away money was added to the standard dictator game, and their experimental results showed that the behavior of the proposers changed. As behavior should remain unchanged in models of outcome-based social preferences, Cooper and Kagel (2016) argue that this may be an experimenter demand-induced effect. By contrast, Krupka and Weber (2013) conducted two sets of experiments to see if norms are affecting these experimental results. They measured the norms of the behaviors possible in variations of the dictator game in their Experiment 1 by reported ratings of social appropriateness for the behavior of giving or taking money. If each participant’s rating was equal to the one that was chosen by the most participants, an additional payment was made.

The norm was measured by Experiment 1, and then the standard version and a “bully” version (that allows the dictator to take money) were administered to another group of participants in Experiment 2. They showed that these differences in the experimental results of Experiment 2 between the two versions can be explained by a model in which utility is obtained from behavior that conforms to the norm using data from the norm in Experiment 1. Thus, for individuals, the model that obtains utility from behavior in line with exogenous norms is promising.

Akerlof and Kranton (2005) define norms as “how people think how they and others should behave.” (p. 12) Norms can vary depending on social contexts, and the identity economics proposed by Akerlof and Kranton (2000, 2005, 2010) focus on social context based on social categories such as gender, race, and religion. Identity is a self-image that an individual belongs to a social category. Experiments in social identity theory suggest that it is easy to create such a self-image. A series of laboratory experiments starting with Tajfel (1970) found that in-group favoritism can be generated by randomizing people into groups that are not related to real economic interests, such as whether they prefer paintings by Klee or Kandinsky, in what is called a minimum group paradigm experiment.

While norms influence subjects from outside the individual economic entity, the author along with his coauthors has been studying the influence of an individual’s internal worldview, such as ethics and values, on behavior. The term “worldview” has various definitions in philosophy and cultural anthropology, but here we use Hiebert’s (2008) definition in anthropology, “the foundational cognitive, affective, and evaluative aspects assumptions and frameworks a group of people makes about the nature of reality which they use to order their lives.” (pp. 25–26) Lee et al. (2014a) provided cases in which worldview beliefs had significant effects on the amount of giving in charity games where the beneficiaries of the dictator game are charities. Lee et al. (2014b) also found that attitudes for altruistic behavior had significant associations with implicit worldviews in questionnaire surveys in Japan and the United States. In this study, the implicit worldview on whether categories or relationships are more important in cognition is measured.

Subsequently, Okuyama et al. (2018) identified significant associations between implicit and explicit worldviews and altruistic behaviors in a Malaysian questionnaire survey that included followers of Islam, Buddhism, Christianity, and Hinduism. They found a significant association where the higher the subjective probability attached to a reincarnation belief, the better the attitudes for pro-environmental protection even after controlling for religion and ethnicity. The possibility of reverse causality from behavioral attitudes to worldviews is unlikely because worldviews are not thought to change after adulthood without major life experiences, and the possibility of omitted variable bias is small because these studies controlled for many variables such as income and education. Hence, these results suggest a causal relationship from worldview to the attitude toward altruistic behavior.

## Virtue and eudaimonia

The discussion in the previous section suggests that behaviors of an individual like giving and donating may increase well-being of the individual as they are consistent with social preferences, norms, and worldviews. For example, Dunn et al.’s (2008) experiment examined the causal effect of behaviors such as giving and donating on happiness. Subjects were given $5 or $20 in the morning and instructed to spend it by 5:00 PM. Two groups were randomly selected: one to spend it on themselves and the other to spend it on others. Spending on others significantly increased happiness, and the amount of money did not make a difference. Another 109 students from the same university were asked to predict which of four conditions in the experiment would make them the happiest. These predictions were doubly wrong. A statistically significant majority of participants predicted that spending money on themselves would make them happier than spending it on others, and that spending $20 would make them happier than spending $5. The students did not correctly predict the determinants of their own happiness. If students are not aware of the increase in happiness that comes from giving, then the tendency to enjoy altruistic preferences and behavior that is consistent with norms and ethics may be formed by actual experience.

The character strengths that contribute to community and society are called virtues. According to Haidt (2006), Martin Seligman, who founded positive psychology in 1998, stated that one of his primary goals was studying the good aspects of people, rather than the pathology and the darker aspects of human nature. His first task with his collaborators was to create a diagnostic manual of virtues. Dahlsgaard et al. (2005) examined virtues in Confucianism, Taoism, Buddhism, Hinduism, Athenian philosophy, Christianity, Judaism, and Islam and found that many virtues are common across diverse cultures and histories. The core virtues are summarized in Table 1 in Dahlsgaard et al. (2005).

Closely related to virtue is eudaimonia, a concept of wellbeing. Annas (2011) answers the question of what virtue and eudaimonia are in a book from the perspective of a philosopher using the analogy of virtue acquisition as the acquisition of skills like playing a musical instrument. Virtue is a perfect tendency that completes all aspects of virtue, such as courage and wisdom, and eudaimonia is the happiness of having such a perfect tendency. While these ideals of virtue and eudaimonia may be useful in ascertaining the correct direction of change in the process of acquiring virtue, it is likely that most human beings do not achieve the ideal of virtue or eudaimonia. For economic research, this paper defines virtue as the tendency to focus on the core virtues shown in Table 1 as strengths, and eudaimonia as the fulfillment of acquiring virtues and the fulfillment of living well using the acquired virtues and abilities to contribute to the community and society. Drawing from this definition, we can interpret that in Dunn et al. (2008), students predicted that utility from their consumption and leisure would have a significant impact on the change in their happiness, but in fact eudaimonia had a significant impact on their changes in happiness.

**Table 1:** Core virtues Source: Dahlsgaard et al. (2005)

Virtue	Description
Courage	Emotional strengths that involve the exercise of will to accomplish goals in the face of opposition, external or internal; examples include bravery, perseverance, and authenticity (honesty)
Justice	Civic strengths that underlie healthy community life; examples include fairness, leadership, citizenship, or teamwork
Humanity	Interpersonal strengths behind taking care of and being a friend to others (Taylor et al., 2000); examples include love and kindness
Temperance	Strengths that protect against excess; examples include forgiveness, humility, prudence, and self-control
Wisdom	Cognitive strengths that entail the acquisition and use of knowledge; examples include creativity, curiosity, judgment, and perspective (providing counsel to others)
Transcendence	Strengths that forge connections to the larger universe and thereby provide meaning; examples include gratitude, hope, and spirituality

## Endogenous preferences and trust

There are many aspects of virtue, and it would be difficult to model the acquisition (or learning) of all aspects of virtue in an economic model, but it would be useful to consider aspects of virtue that are particularly important to economics in an economic model. From this perspective, the endogenous time preference model of Becker and Mulligan (1997) shows that the greater the human capital that enables people to imagine the future more vividly, the larger the time discount factor. In this model, the virtue of patience, considered to be one of the virtues of temperance in such a model, can be taken to be a state in which the time discount factor is equal to one, which is a fair evaluation of one’s current and future utility. Then when a time discount factor smaller than one becomes larger, this can be interpreted as learning of the virtue of patience.

Alan and Ertac (2018) conducted a field experiment in a Turkish elementary school with an intervention to promote perseverance in third and fourth graders. They developed an educational program of intervention based on the theoretical model of Becker and Mulligan (1997). They measured children’s patience in an experiment that measured the time discount factor. The effects of the intervention were measured in a randomized controlled trial, and the children who received the intervention made more patient decisions in the experiment, an effect that persisted 3 years after the intervention. This indicates that schooling can influence human capital to learn virtues through changes in time preference, and that interventions can also influence this learning.

Generalized trust is often used in the study of social capital in economics, and in the World Values Survey, the question “Generally speaking, would you say that most people can be trusted, or that you can’t be too careful in dealing with people?” has been often used to measure general trust.

Algan et al. (2013) examined the effects of educational methods on general trust in various data, using “teacher-led lectures” as a vertical educational method and “group learning among students” as a horizontal educational method in schools. They found causal evidence of a positive effect of group learning on general trust. Ito et al. (2020) studied the association of educational methods in Japanese elementary schools with trust, reciprocity, and altruism in adulthood. Group learning was positively and significantly associated with trust, reciprocity, and altruism. If we interpret trust as the belief that others have kind intentions toward you in Rabin’s (1993) model, then an interpretation of these results that is consistent with this model would be that an increase in social capital in the form of trust led to an increase in reciprocity and altruism.

## Leadership

In Table 1, leadership is listed as an example of the virtue of justice. The importance of leadership has been pointed out in business administration, and this may be partly because leadership is important for community mechanisms to work well in companies. As an example of a local community, the author interviewed a group called Group Schole, which is active in Senboku New Town, Osaka prefecture, where the population is aging, in 2015. The following figures are current at the time of the interview. At the time, the group had about 60 home courses and almost 280 members, with an average age exceeding 66 years.

The founder, Ms. Kazuko Toshiyasu (71 years old at the time of the interview), started the program in 1996. The members open their homes and teach their specialties such as cooking, jazz, chanson, and mahjong for free, and after the class, they collect 500 yen per person for teatime.

Many people have heard about Group Schole and have started similar groups, but they have not been able to stick with them. When the leader is criticized by members who are dissatisfied with something, they quit. Why was Group Schole able to keep going when the leader of the group was criticized in the same way. It seemed important that the group of leaders were able to support each other, because Ms. Toshiyasu did not remain the only leader of the group but nurtured the leaders by replacing them with other leaders.

From the perspective of creating a community with diverse members, we would like to focus on servant leadership in which the leader serves each member so that he/she can contribute to the objective of the community and grow rather than top-down leadership. Robert Greenleaf (1904–1990) coined the term Servant Leadership in English in an essay included in Greenleaf (1977) which was first published in 1970. Spears (1995) and van Direndonck (2011) list the characteristics of servant leadership as listening, healing, empathy, stewardship, commitment to people’s growth, etc. It would appear that Ms. Toshiyasu’s leadership possessed servant leadership characteristics such as commitment to people’s growth.

Here, van Direndonck (2011) states that stewardship “is the willingness to take responsibility for the larger institution and to go for service instead of control and self-interest.” Therefore, leaders “should act not only as caretakers but also as role models for others.” (p.1294) By setting the right example, leaders can stimulate others to act in the common interest. Such leading by example has been modeled in economics by Hermalin (1998) and Kobayashi and Suehiro (2005).

## Perspective taking and community mechanisms

Regarding the perspective in the examples of the virtue of wisdom in Table 1, many of the cases of criticism that disrupt communities, such as those introduced in the previous section, could be avoided or expressed in a constructive way if the criticizing members could think from the perspective (or viewpoint) of the one being criticized. Because of a cognitive bias that Kahneman (2011) called What You See Is All There Is (WYSIATI), we are aware of our own pain of contributing to the community, but we will never be able to fully understand the pain of others. It seems necessary for us to know we can never fully understand the pain of others and humbly try to understand it.

In terms of modeling taking another person’s point of view, Kaneko and Kline (2015) developed the inductive game theory proposed by Kaneko and Matsui (1999) into a theory of games in which two people occasionally engage in role switching. Takeuchi et al. (2015) found in an experiment that more cooperative behavior was obtained when there was role switching than when there was not, as predicted by this theory.

The concept of general will in Rousseau’s Theory of the Social Contract is based on his belief that if the members of a society are completely free to leave their own interests and positions and take the perspective of all other members, they will all unanimously have the general will, which is the correct will that aims at the common good. In this sense, general will is related to the change of perspective from oneself to others.

Relying on Sakai’s (2015) model, we discuss Rousseau’s general will and collective decision-making through voting. In the model, *i* = {1,2,…, n} is the set of voters, a closed interval of [0,1] is the set of possible choices for an issue, and each voter *i* prefers *t*_i_ as the best choice and has a preference *R*(*t*_i_) with Euclidean distance. That is

$$xR\left( {t_{i} } \right)y \Leftrightarrow \left| {x - t_{i} } \right| \le \left| {y - t_{i} } \right|{ }\forall { }i \in \left[ {0,1} \right]$$

where *i* ∈ [0,1]. Suppose that voting is done on a finite subset of agendas in [0,1].

Sakai (2015) introduced the sympathy condition that when these preferences are arranged in a horizontal line segment, the voter on the leftmost side weakly prefers the best option of the voter on the rightmost side over zero, and the voter on the rightmost side weakly prefers the best option of the voter on the leftmost side over one. Sakai (2015) proved that given standard assumptions in the field, such as *n* being odd, if the sympathy condition holds, then the Borda winner coincides with the Condorcet winner or the Condorcet second winner. He also proved that when the number of agendas becomes infinitely large, the Borda winner converges to the best option of the median voter.

To consider the relationship between these results and the general will, suppose, as an example, that a country is trying to decide by voting how much international cooperation it will provide in combating climate change. To correspond with the political left and right, the choices here are zero for full cooperation and one for complete noncooperation. Imagine that the best options from the selfish preferences, which consider the position of business owners and workers who would benefit from new oil drilling on public lands, or from the advancement of offshore wind power, do not satisfy the sympathy condition. The best option for everyone with this preference corresponds to Rousseau’s private will. In the case of voting based on private will, the Borda winner and Condorcet winner do not coincide except in special cases.

By contrast, Rousseau assumes that each voter is not a slave to private will, meaning that when they are completely free, all will unanimously choose the same option, and this option is called general will. Let us consider a case where people would vote without being completely free in this sense, but with sufficient sympathy for the positions and roles of other voters in society. With an assumption that the preferences in this case satisfy the sympathy condition, the voting approximates the general will in two senses. First, the Borda winner coincides with the Condorcet winner or the Condorcet second winner. Second, the Borda winner coincides with the Condorcet winner if the number of agendas is increased infinitely.

Immanuel Kant advocated an ethical approach called deontology, in which obligations from general moral laws, such that everyone should be valued as an end rather than a means, should be fulfilled with pure motives. Everyone’s ethical judgment is thus based on the purity of their own motives. Because other people’s motives cannot be judged, for evaluating laws and institutions, Kant advocates social contract based on freedom. Sandel (2009) argued that Kant’s social contract is based on a hypothetical agreement that the whole nation can agree to, even if they do not actually agree to it. Sandel (2009) argues that Rawls’ (1971) theory of justice is an attempt to answer what Kant’s hypothetical contract would look like.

Rawls holds that if people choose the principles of this hypothetical contract under a “veil of ignorance,” leaving aside their own position in real life, two kinds of principles of justice will be chosen. First, basic freedoms such as freedom of speech and religion will be granted equally to all people. The second principle is to allow only social and economic inequalities that benefit the most disadvantaged in society. The maximin function is used to express the second principle in terms of a social welfare function.

Kameda et al. (2016) showed that in a task in which experimental participants distribute payoffs to three other people and in a task in which they choose a lottery for themselves, participants’ distributive choices closely matched their risk preferences of lotteries. Participants who chose maximin when distributing to others also often made choices that maximize the gain in the smallest gain case in the task in which they choose a lottery for themselves. On the other hand, participants who favored the largest total distributions preferred riskier but more profitable lotteries. Participants, including those who did not choose maximin, tended to show interest in the lowest payoff in both tasks. The right temporoparietal junction was activated when participants showed interest in the lowest payoff. This region has been shown to be associated with perspective taking. From these results, Kameda et al. (2016) conjecture that perspective taking is a key to understand the linkage between distributive and risky decisions: “perspective taking here means mentally stimulating a different standpoint (…)-how one would feel if placed in situations that differ physically or temporally (“other/future”) from one’s immediate environment (“myself/now”).” (p. 11818) Many participants were able to make decisions by paying attention to the worst case of others without being instructed to wear the “veil of ignorance.”

## Normative economics and the community mechanism

There are two major branches of economics: positive economics, which deals with the ethical- and value-neutral scientific question of how resources are allocated, and normative economics, which deals with the question of how resources should be allocated, which cannot be ethical- and value-neutral. The purpose of this section is to discuss normative economics with an emphasis on the community mechanism.

### Analytical framework introducing virtue ethics

Normative ethics is the study of theorizing people’s ethical views. There are three major approaches in normative ethics: consequentialism, deontology, and virtue ethics. Consequentialism is an approach of making ethical judgments based only on the consequences of an action, not on the motivation for the action or the decision-making process. Examples are utilitarianism and welfarism, which are commonly used in economics. Deontology is an approach that emphasizes moral obligations. A representative example is Immanuel Kant’s theory previously mentioned. Virtue ethics is an approach that emphasizes virtue. This can be seen as an ethical view that considers it good to contribute to the community by acquiring virtue, and that eudaimonia as a sense of fulfillment from contribution is a concept of happiness in this approach. Since virtue is important for the community mechanism to work well, it would be desirable to introduce a virtue ethics approach to normative ethics.

Kaplow and Shavell (2011) showed that the Pareto principle does not hold true when an ethical theory other than welfarism is introduced to evaluate resource allocation. For example, Sen’s (1970) liberal paradox that the Pareto principle is incompatible with the ethical theory of liberalism. Based on a model in which preferences are exogenously given, it would be difficult to escape the conclusion that other ethical theories should not be introduced because there would have to be very strong reasons for using an ethical theory other than welfarism to oppose a change in the distribution of resources that is preferred by all people.

However, if we think in terms of an endogenous preference model, in which preferences vary with policies, etc., then an evaluation of “preferred by all” would be less powerful because this evaluation is done by some chosen preference orderings among many preferences that vary endogenously with economic conditions, thus introducing other ethical theories in evaluation will also be more convincing. For example, even if each person’s utility would strictly increase if all people consumed addictive drugs, if addictive preferences are less desirable than nonaddictive preferences from the perspective of virtue ethics, it is unlikely that everyone’s consumption of drugs would be socially desirable. From this perspective, the results of Kaplow and Shavell (2001) can be interpreted as showing that the Pareto principle is not a weak ethic when other ethical theories are combined with welfarism even though it is weak within welfarism.

Based on this idea, Bhatt et al. (2017) proposed an analytical framework that introduces virtue ethics as well as pure welfare principles into models with endogenous preferences. Bhatt et al. (2015) applied this analytical framework to advocate the principle of “learning to unconditionally love,” in which utility-based welfarism is balanced with virtue learning toward the moral obligation to unconditionally love.

In Bhatt et al.’s (2017) analytical framework for models with endogenous preferences, welfarism and virtue ethics are in balance. First, to use welfarism to evaluate resource allocations using standard tools such as Pareto efficiency and social welfare functions, it is not possible to use preferences that change endogenously within the economy as a yardstick for the evaluation. So, some exogenous preferences need to be chosen. As an example, Pollak (2013) distinguished between conditional preference ordering, which changes depending on conditions such as past consumption, and unconditional preference ordering, which takes into account changes in preferences before such changes occur, and proposed that unconditional preference ordering be used for evaluation based on welfarism. Second, they use Sen’s (1974, 1977) meta-preference idea that there can be preferences that are ethically more favorable to society to introduce virtue ethics.

When virtue ethics is introduced, the Weak Pareto Principle will be violated and require modification:

Modified Weak Pareto Principle: Given two allocations of resources* x* and *y*, if everyone strictly prefers *x* to *y*, then *x* should be evaluated to be better for society as long as *x* is not evaluated to be worse than *y* by other ethically relevant factors.

This modification of adding “as long as *x* is not evaluated to be worse than *y* by other ethically relevant factors” to the Weak Pareto Principle is an application of Temkin’s (2011) concept to endogenous preference models by Bhatt et al., (2015, 2017). Bhatt et al. (2017) use meta-preference for the introduction of the virtue ethics principle.

The Principle of Virtue Ethics: Given two allocations *x* and *y*, if at least one person’s conditional preference ordering is strictly better in terms of virtue ethics and everyone else’s conditional preference ordering is at least as good in terms of virtue ethics in *x* than in *y*, then *x* should be evaluated to be better than *y* for society.

As with the Weak Pareto Principle, a modification is needed to consider welfarism at the same time as virtue ethics.

The Modified Criterion of Virtue Ethics: Given two allocations *x* and *y*, if at least one person’s conditional preference ordering is strictly better in terms of virtue ethics and everyone else’s conditional preference ordering is at least as good in terms of virtue ethics in *x* than in *y*, then *x* should be evaluated to be better than *y* for society as long as *x* is not evaluated to be worse than *y* in terms of other ethically relevant factors.

Under these preparations, with *x* as a resource allocation, Bhatt et al. (2017) propose adding two functions to a standard social welfare function *W(x)* that satisfies the Weak Pareto Principle: a moral evaluation function *M(x)* that satisfies the Virtue Ethical Principle and evaluates the nature of endogenous preferences in each resource allocation, and a social objective function *S(W(x), M(s))* that satisfies the modified weak Pareto principle and the modified weak virtue ethics principle, and propose to analyze the optimal policy as the policy that maximizes the social objective function.

As an application of such an analytical framework, Bhatt et al. (2015) developed an economic model (based on Mulligan, 1997) in which parents’ altruism toward their children increases when they spend more time with them. When virtue ethics is introduced as well as welfarism, which emphasizes utility, optimal policy changes from prioritizing GDP to considering the deepening of bonds through time spent at home. Their numerical example shows that the introduction of virtue ethics does not necessarily lead to an increase in government intervention.

### Application to childcare outsourcing

In childcare, the community mechanism among the three groups of children, parents, and childcare center staff is important. When a university or a hospital uses a consignment contract for childcare services, there is a risk that the quality of childcare will rapidly decline if this community is not protected.

Ogaki et al. (2022) used qualitative research methods, primarily interviews, to investigate the consign contracts of childcare services at two universities and found that biddings and open calls are not always desirable for economic efficiency and fairness, when considering the functioning of the community. In some cases, it is preferable not to conduct biddings or open calls but to continue to enter negotiated contracts with the childcare organization in which parents participate in the management of childcare and conduct audits and evaluation of the quality of childcare on a regular basis.

Biddings and open calls may also not be desirable for economic efficiency because economically efficient childcare requires relation-specific investment by childcare workers and parents. It is desirable to provide stable employment and employment conditions so as not to discourage the investment of childcare workers. In addition, in order not to discourage parents’ investment, there are advantages to organizations such as nonprofit organizations where parents participate in the childcare center’s management.

From the principle of equality of opportunity, bidding and open calls are not necessarily desirable. This is because it is desirable for the sake of equality to reward past relation-specific investments. Given the fact that young children’s cognitive abilities are inadequate for making important market decisions alone, ethical perspectives other than economic efficiency and fairness need to be considered. From the perspective of virtue ethics, if the personal development of the child is important and the bonds of the childcare community are sufficiently strong, then bidding and open calls are not desirable.

## Conclusion

In this paper, we defined the community mechanism as a mechanism for allocating resources by which at least one person proposes voluntary cooperation, and the proposal is not rejected. In times of crisis, isolation is dangerous, so the community mechanism is likely to become increasingly important. If there is a win–win situation for the community mechanism, it will start to work, but if there is awareness of eudaimonia from the experience of learning virtue and contributing to the community using virtue, further development will be possible. When evaluating what is a good policy or action, it would be beneficial to also consider virtue ethics theory to avoid drawing conclusions that focus only on economic efficiency and harm the functioning of community mechanisms.

One future research direction is to deepen our understanding of the relationship between trust and the community mechanism. The main project of the OECD Trustlab Project, as described by Murtin et al. (2018), conducts an internationally comparable online experiment and survey in each country for a representative sample (in the sense of representativeness for sex, age, and income) for 1000 people or more individuals. The cross-sectional data for the seven countries were collected by 2019.

By contrast, Japan collected panel data for the first time in the world in the main project of Trustlab: Wave 1 in January–February 2020, Wave 2 in June–July 2020, and Wave 3 in September–October 2021.^6^ Preliminary results for Waves 1 and 2 show that trust, altruism, and reciprocity are positively correlated, with large movements in both positive and negative directions when they change. Since there are correlations among the variables, the changes do not seem measurement errors. This is consistent with the fact that in some cases, family ties were strengthened while in other cases, domestic violence was aggravated, or divorce occurred because the family spent more time together due to telework that was increased after COVID-19 struck. However, more detailed analysis is needed in the future to investigate the reason for the changes.

Based on the idea that the community mechanism is important in the era of crisis, another plan of a study is to conduct online experiments and interviews with members of the “Global Republic,” which was created in April 2021 by Ms. Yukiko Minami on an audio SNS and is rapidly growing with more than 8000 members as of March 2022.^7^ The Global Republic’s main slogan is “Community changes the world,” which is consistent with the increased importance of the community mechanism at times of crisis. Its code of conduct lists “One for All; All for One” first, which emphasizes both the importance of each one to contribute to the community and that of the community to value each member.

One direction for future research is theoretical and empirical research on trust as a moral value proposed by Uslaner (2002, 2008) rather than trust as the rational belief of other people’s trustworthiness as in many economic theories. Uslaner (2002, p. 1) argues that “[t]rusting strangers means accepting them into our ‘moral community’. Strangers may look different from us; they may have different ideologies or religions. But we believe that there is an underlying commonality of values.” This can be thought of as an investment of trust by Robinson, for example, in the story of Robinson and Friday, given the change in Friday’s moral views. This is because when people are trusted, they seem more willing to reciprocate the trust. Trust as a moral value is not a rational prediction from experience but based on a view of human beings that we have commonality of values deep in our hearts as our conscience even though we also have many differences. We now tend to interact with many people with different worldviews and religions with persistent divides in some beliefs. We may still be able to cooperate and benefit from diversity, as in Mihailov and Ogaki (2021).

Another direction of future research is to deepen our understanding of how preferences are formed in relation to virtue ethics. Akabayashi et al. (2014) developed an experimental method for parent–child pairs to study how parenting may affect children’s time preferences. An ongoing research project has collected panel data of parent–child pairs in Japan and the United States.^8^ Sasaki et al. (2017) used hypothetical questions in Internet surveys across five countries to study how education may affect altruistic preferences.

## Footnotes

1 Financial mistakes include suboptimal use of credit card balance transfer offers and excess interest and fee payments (Agarwal et al., 2009).

2 Ogaki (2003) gives an example of an aggregation theorem with a review of others.

3 If one person accepts the proposal, then the community mechanism works for two persons. However, if a group decision is made by voting to reject the proposal, then the community mechanism fails to work for the proposal, even when a person votes to accept the proposal.

4 Similarly, repeated encounters are not required for this definition, even though long-term relationships can be useful for further developments of the community mechanism.

5 Bowles and Polanía-Reyes (2012) provide a survey of experimental results of economic incentives and social preferences and theories based on social preferences (which also include norms in the terminology of the present paper) that can be interpreted as a study of the effects of various combinations of the market and community mechanisms.

6 This research project is briefly explained by Hanaki et al. (2022).

7 This is a joint research plan with Masami Aki, Nobuyuki Hanaki, and Yukihiko Funaki.

8 This is a joint research project with Hideo Akabayashi, Vipul Bhatt, Ryousuke Nakamamura, and Teruyuki Tamura.
